# Alcohol Use in Adolescence and Later Working Memory: Findings From a Large Population-Based Birth Cohort

**DOI:** 10.1093/alcalc/agx113

**Published:** 2018-01-10

**Authors:** Liam Mahedy, Matt Field, Suzanne Gage, Gemma Hammerton, Jon Heron, Matt Hickman, Marcus R Munafò

**Affiliations:** 1 Population Health Sciences, Bristol Medical School, University of Bristol, Oakfield Grove, Bristol BS8 2BN, UK; 2 Department of Psychological Sciences, University of Liverpool, Bedford Street South, Liverpool L69 7ZA, UK; 3 UK Centre for Tobacco and Alcohol Studies, University of Liverpool, Liverpool, UK; 4 School of Experimental Psychology, University of Bristol, Priory Road, Bristol, BS8 1TU, UK; 5 MRC Integrative Epidemiology Unit (IEU), University of Bristol, Oakfield Grove, Bristol BS8 2BN, UK; 6 UK Centre for Tobacco and Alcohol Studies, School of Experimental Psychology, University of Bristol, Priory Road, Bristol BS8 ITU, UK

## Abstract

**Aims:**

The study aimed to examine the association between adolescent alcohol use and working memory (WM) using a large population sample.

**Methods:**

Data from the Avon Longitudinal Study of Parents and Children were used to investigate the association between alcohol use at age 15 years and WM 3 years later, assessed using the *N*-back task (*N* ~ 3300). A three-category ordinal variable captured mutually exclusive alcohol groupings ranging in order of severity (i.e. low alcohol users, frequent drinkers and frequent/binge drinkers). Differential dropout was accounted for using multiple imputation and inverse probability weighting. Adjustment was made for potential confounders.

**Results:**

There was evidence of an association between frequent/binge drinking (compared to the low alcohol group) and poorer performance on the 3-back task after adjusting for sociodemographic confounding variables, WM at age 11 years, and experience of a head injury/unconsciousness before age 11 years (*β* = −0.23, 95% CI = −0.37 to −0.09, *P* = 0.001). However, this association was attenuated (*β* = −0.12, 95% CI = −0.27 to 0.03, *P* = 0.11) when further adjusted for baseline measures of weekly cigarette tobacco and cannabis use. Weaker associations were found for the less demanding 2-back task. We found no evidence to suggest frequent drinking was associated with performance on either task.

**Conclusions:**

We found weak evidence of an association between sustained heavy alcohol use in mid-adolescence and impaired WM 3 years later. Although we cannot fully rule out the possibility of reverse causation, several potential confounding variables were included to address the directionality of the relationship between WM and alcohol use problems.

## INTRODUCTION

Alcohol consumption during adolescence is a major public health concern, in particular because the brain is still developing and undergoing considerable structural and functional changes (De Bellis *et al.*, 2000). One area of research that has received considerable attention is the association between alcohol use and working memory (WM) performance. WM is critical to higher order cognitive functioning, such as decision making and planning (Miller and Cohen, 2001) and deficits in WM make it more difficult to respond in a controlled and planned manner to alcohol stimuli (Grenard *et al.*, 2008; Peeters *et al.*, 2012). Furthermore, WM may be more susceptible to damage from heavy alcohol use during adolescence than in adulthood (De Bellis *et al.*, 2000) as it is not fully developed until young adulthood (De Luca *et al.*, 2003; Boelema *et al.*, 2014).

Research using both animal models and human data has provided evidence to suggest a negative association between alcohol use and WM during adolescence. For example, research in animal models has demonstrated lasting consequences of adolescent exposure to alcohol, including alterations in later WM performance (White *et al.*, 2000; Risher *et al.*, 2013). Human brain imaging studies have identified neural functioning correlates of adolescent heavy drinking. Studies using co-twin designs have found that amygdala deficits (Wilson *et al.*, 2015), smaller orbitofrontal cortex volumes and diminished quality of decision making (Malone *et al.*, 2014) are associated with adolescent alcohol use. Brain imaging studies examining singletons have found that young people with alcohol use disorders have smaller hippocampal volumes (De Bellis *et al.*, 2000; Nagel *et al.*, 2005), prefrontal abnormalities at both the structural (De Bellis *et al.*, 2005) and functional levels (Tapert *et al.*, 2004), and damage to the frontal lobe (Crews *et al.*, 2007). Taken together, these findings suggest that alcohol use during adolescence may be associated with risks for neurocognitive deficits, however, the direction of the association is not clear.

Studies that have examined the prospective association between alcohol use and WM functioning using both brain imaging and neuropsychological study designs have largely revealed mixed findings. Some studies found that alcohol use preceded WM functioning (Squeglia *et al.*, 2009; Squeglia *et al.*, 2012; Peeters *et al.*, 2014); while others found evidence for the opposite direction (Peeters *et al.*, 2014; Peeters *et al.*, 2015), in that, adolescents with poor WM may be at increased risk of developing alcohol problems.

Evidence from the limited number of prospective community samples that have examined this relationship has shown an equally conflicting pattern of results. For example, Boelema *et al.* (2015) found no evidence of an association between heavy drinking in adolescence and maturation of executive functioning. The discrepancies in the literature could be due to a number of factors, including (a) sample size, (b) study design (high-risk vs community-based samples), (c) the alcohol use phenotype (i.e. binging vs frequency), (d) lack of control for potentially relevant confounding factors and (e) different follow-up periods.

While acknowledging that it is possible that deficits in cognitive functioning could precede and influence alcohol use, this study sought to expand on previous research by using a large UK birth cohort to examine the possibility that the neurotoxic effects of alcohol during this sensitive developmental period may impact on later cognitive functioning. Focusing on this one potential pathway, we hypothesized that sustained heavy drinking, defined as frequent and binge drinking at age 15 years (peak incidence for alcohol use Melotti *et al.*, 2013), would be adversely associated with WM at age 18 years (as WM matures in late adolescence) (De Luca *et al.*, 2003; Boelema *et al.*, 2014) while controlling for potentially relevant confounding factors, including a measure of WM assessed prior to the onset of alcohol use.

## METHOD

### Participants and procedure

We used data from the Avon Longitudinal Study of Parents and Children (ALSPAC), an ongoing population-based study that contains a wide range of phenotypic and environmental measures, genetic information and linkage to health and administrative records. A fully searchable data dictionary is available on the study’s website (www.bris.ac.uk/alspac/researchers/data-access/data-dictionary/). Approval for the study was obtained from the ALSPAC Ethics and Law Committee and the Local Research Ethics Committees. All pregnant women residing in the former Avon Health Authority in the south-west of England between 1 April 1991 and December 1992 were eligible for the study (Phase I consisted of *n* = 14,541). Of the 13,978 offspring alive at 1 year, a small number of participants withdrew from the study (*n* = 24), leaving a starting sample of 13,954. Detailed information about ALSPAC is available online www.bris.ac.uk/alspac and in the cohort profiles (Boyd *et al.*, 2013; Fraser *et al.*, 2013). A detailed overview of our study population, including attrition at the different measurement occasions, is shown in the Supplementary Material Fig. S1.

### Measures

A timeline for data collection is shown in Supplementary Material Fig. S2.

#### Exposures: adolescent alcohol use

At ~15 years of age (*M* = 15.5; SD = 0.35), participants completed a computer-based session at a research clinic, which included questions regarding drinking frequency and binge drinking. The following two binary variables (present vs absent), as previously defined (Melotti *et al.*, 2013), captured alcohol involvement: (a) frequent drinking (≥20 times in the previous 6 months) and (b) regular binge drinking (consuming more than five drinks in any 24-h period on ≥20 occasions in the previous 2 years), which adapts a common definition of binge drinking (Masten *et al.*, 2008). An ordinal variable capturing three mutually exclusive groups was created by combining these two measures. Groups consisted of participants who did not meet either criterion, *n* = 3525 (78.9%), from here on referred to as the ‘low’ alcohol group, participants who were frequent drinkers only, *n* = 480 (10.8%), and participants who were binge and frequent drinkers *n* = 461 (10.3%). High thresholds for alcohol use were used to capture the extreme end of consumption as it has been suggested that the amount of alcohol consumed in community samples might be too low to negatively influence the development of WM (Khurana *et al.*, 2013). For all analyses, the low alcohol group was taken as the reference group.

#### Outcome: working memory

A computerized version of the *N*-back task, including both 2- and 3-back conditions (*N* = 4827), was used to assess WM at the age 18 years research clinic (*M* = 17 years 10 months; SD = 5 months). The *N*-back task, originally introduced by Kirchner (Kirchner, 1958) is widely used to measure WM (Wardle *et al.*, 2013; Rossi *et al.*, 2016), and has been shown to activate prefrontal cortex (PFC) areas (Cohen *et al.*, 1997). Despite being frequently used in brain imaging studies, there have been few psychometric studies of the *N*-back task. In general, the studies that have examined its psychometric properties have reported reliability coefficients >0.70 (Schmiedek *et al.*, 2009; Jaeggi *et al.*, 2010; Unsworth, 2010).

Four metrics were examined for both the 2- and 3-back conditions: (a) hits, or the percentage of matching numbers correctly identified as matches, (b) false alarms, or the percentage of non-matching numbers incorrectly identified as matches, (c) discriminability index, *d*′, which is a signal-detection metric that takes into account both hits and false alarms to derive an overall estimate of signal-detection ability, see McNicol (1972) and (d) median reaction times for hits and false alarms, as an indicator of processing efficiency. A measure of *d*′ was chosen as the primary outcome measure given it is an overall estimate. The remaining indices were examined for descriptive purposes. High scores on number of hits indicated more accurate identification, while high scores on false alarms indicated less accurate identification. High scores on *d*′, therefore, indicated a greater ability to distinguish signals from noise.

As we were interested in examining possible enduring effects of alcohol use on WM performance, WM was assessed at age 18 years as it generally shown that WM matures in late adolescence (De Luca *et al.*, 2003; Boelema *et al.*, 2014). Participants were excluded if they did not provide any responses (*n* = 373 for the 2-back task; *n* = 320 for the 3-back task). In total, *n* = 3141 participants completed both versions of the task, while *n* = 3351 participants had available data for the 2-back task and *n* = 3319 had available data for the 3-back version.

### Potential confounders

Given the complicated confounding structure, potential confounding variables were included in three steps. First, we examined an unadjusted model (Model 1). Second, a number of sociodemographic measures were considered to be potential confounders of the relationship between alcohol and WM in adolescence (Model 2). These comprised of established risk factors for WM performance for which we felt the assumption of a causal predictive relationship with earlier alcohol use could be justified. Adjustment was made for a number of potential time-invariant confounding variables during pregnancy. These included: income (quintiles), maternal education (<O level: indicating no qualification; O level: indicating completion of school examinations at age 16; and >O level: indicating completion of college or university education at or after age 18), socioeconomic position (SEP, grouped into four categories: (a) unskilled or semiskilled manual; (b) skilled manual or non-manual; (c) managerial and technical and (d) professional), parity (first, second, ≥third children), housing tenure (mortgaged, subsidized renting and private renting), sex and maternal smoking during first trimester in pregnancy (yes/no).

Third, WM at approximately age 11 years and experience of a head injury/unconsciousness up to age 11 years were included (Model 3). A computerized version of the Counting Span task (Case *et al.*, 1982) was included at approximately age 11 years (*M* = 10 years, 8 months, SD = 3 months) to assess WM performance during a focus clinic. A span score was based on the number of correctly recalled sets (maximum score of 5 in increments of 0.5). Further detail is provided in the Supplementary Material. A measure of head injury/unconsciousness was also included. Since adolescents who have experienced head injury perform poorly compared with age-matched peers (Newsome *et al.*, 2007), we included participants who experienced head injury/unconsciousness before the age of 11 years, *n* = 113 (3.4%). The inclusion of both measures, prior to the onset of alcohol initiation, helps to remove the possibility that deficits in WM performance precedes alcohol use, thereby allowing for the temporal order between alcohol use and later WM to be established.

Finally, weekly cigarette smoking and cannabis use at age 15 years (assessed during the same clinic assessment as the alcohol measures) were included (Model 4). Weekly cigarette smoking, assessed using the question ‘do you smoke every week’ (*n* = 181/2659), was included because of evidence suggesting that smoking is associated with cognitive function (Loughead *et al.*, 2009). Cannabis use in the past 12 months, assessed using the question ‘has used or taken cannabis in the past 12 months’ (*n* = 442/2649), was included as evidence suggests that engagement in cannabis use often display deficits in neurocognitive function (Henderson *et al.*, 1999).

### Statistical methods

A series of univariable and multivariable linear regression models were conducted to examine the association between each of the alcohol exposures and the 2- and 3-back outcome measures. Models unadjusted and adjusted for potential confounding variables were examined. *d*′ was chosen *a priori* as the primary outcome as it captures overall signal detection. Number of hits, false alarms, and reaction time for hits and false alarms were used as secondary outcomes allowing for specific effects to be examined. Standardized regression coefficients with 95% confidence intervals were used and can be interpreted as a change in the exposure associated with a one standard deviation change in WM performance assessed using the *d*′ measure (i.e. our primary outcome). All standardized scores are normalized to have a mean of 0 and a standard deviation of 1, and thus regression coefficients can be interpreted as effect sizes.

### Attrition

Since using complete case analysis can result in biased estimates (Sterne *et al.*, 2009), we examined possible effects of missing data using a combination of multiple imputation and inverse probability weighting (MI/IPW) (Seaman *et al.*, 2012). In the first step, MI was based on the 3351 participants who had information on the 2-back task, and 3319 participants who had information on the 3-back task. The imputation model contained performance on both versions of the WM task, alcohol exposure variables, and potential confounding variables, as well as a number of additional auxiliary variables known to be related to missingness. Fifty datasets by 10 cycles of regression were generated.

In the second step, IPW was performed. Estimates of prevalence and associations were weighted to account for probabilities of non-response to attending the clinic. Further information is provided in the Supplementary Material. All analyses were conducted using Stata version 14. Results using weighted estimates are reported as the main results. Supplementary Material Table S1 shows strong evidence of a relationship between sociodemographic variables, a measure of WM at age 11 years, cigarette smoking, cannabis use, and alcohol use at age 15 years, and missing data on both conditions of the WM task. Furthermore, individuals who attended the clinic at age 18 years but who did not complete either of the *N*-back tasks were more likely to be involved in frequent and binge drinking at the earlier age of 15 years (Supplementary Material Table S2).

### Sensitivity analyses

As WM is developing across childhood and adolescence, a measure of WM performance, assessed at age 8 years, was included to provide consistency across the findings. The backward digit span task was assessed as part of an in-person standardized assessment of cognitive ability at age 8 years. The backward measure, which requires storage and manipulation of the information prior to recall, is thought to tap into WM capacity (Alloway *et al.*, 2006). Further information on the measure is provided in Supplementary Material. Models using complete cases were included to assess the impact of missing data.

## RESULTS

### Descriptive results

WM performance at age 18: Overall, participants performed better on the 2-back task compared to the 3-back task using all four metrics (Supplementary Material). In terms of WM performance for the specific alcohol groups, frequent and binge drinkers performed worse on the 2-back task, assessed using the *d*′, (*M* = 1.70, SD = 1.36) compared to the low alcohol group (*M* = 1.84, SD = 1.24) and the frequent drinking only group (*M* = 1.92, SD = 1.19). A similar pattern was observed for performance on the 3-back task with frequent and binge drinkers performing worse (*M* = 1.02, SD = 1.04) compared to the low alcohol (*M* = 1.19, SD = 1.03) and frequent drinking groups (*M* = 1.28, SD = 1.04).

Associations between alcohol use at age 15 years and potential confounding variables are presented in Supplementary Material Table S3.

### Univariable and multivariable linear regression

Table 1 presents the associations between alcohol use at age 15 years and 2-back task performance (assessed using *d*′) at age 18 years. There was insufficient evidence to suggest an association between frequent drinkers compared to the low alcohol group and WM performance. There was evidence in the unadjusted models that frequent and binge drinking (*β* = −0.17, 95% CI = −0.32 to −0.03, *P* = 0.02) was associated with WM performance compared to the low alcohol group. However, this association was attenuated when adjusting for sociodemographic variables, WM at age 11 years and participants who had a head injury/unconsciousness before age 11 years, weekly cigarette smoking, and cannabis use assessed at age 15 years (*β* = −0.05, 95% CI = −0.21 to 0.10, *P* = 0.50).

**Table 1.** Associations between alcohol use at age 15 years and *d′* at age 18 years for the 2-back task (*n* = 3351) in 50 multiply imputed datasets (standardized coefficients)

	*n* (%)	Model 1		Model 2		Model 3		Model 4	
		*β* (95% CI)	*P*	*β* (95% CI)	*P*	*β* (95% CI)	*P*	*β* (95% CI)	*P*
Low alcohol use		ref		ref		ref		ref	
Frequent drinking only	397 (11.7)	−0.00 (−0.15, 0.14)	0.96	−0.03 (−0.16, 0.10)	0.76	−0.02 (−0.15, 0.11)	0.72	0.03 (−0.11, 0.17)	0.67
Frequent and binge	368 (10.8)	−0.17 (0.32, −0.03)	0.02	−0.15 (−0.30, 0.00)	0.05	−0.16 (−0.30, −0.01)	0.03	−0.05 (−0.21, 0.10)	0.50

Model 1: unadjusted; Model 2: adjusted for sex, income, social economic position, maternal education, housing tenure, parity and maternal smoking in pregnancy; Model 3: further adjusted for working memory assessed at approximately age 11 years, and head injury/unconsciousness up to age 11 years; and Model 4: further adjusted for young person cigarette and cannabis use assessed at age 15 years.

Table 2 presents associations between alcohol use at age 15 years and 3-back task performance at age 18 years. In a similar finding to the 2-back task, there was insufficient evidence to suggest an association between frequent drinking compared to the low alcohol group and WM performance assessed using *d*′. There was however strong evidence that frequent and binge drinking compared to the low alcohol group was associated with WM performance: unadjusted model (*β* = −0.25, 95% CI = −0.39 to −0.11, *P* < 0.001), model adjusted for sociodemographic confounders (*β* = −0.22, 95% CI = −0.36 to 0.08, *P* = 0.001), model further adjusted for WM at age 11 years and participants who had a head injury/unconsciousness before age 11 years (*β* = −0.23, 95% CI = −0.37 to −0.09, *P* = 0.001). However, this association was attenuated when further adjusted for weekly cigarette smoking and cannabis use at age 15 years (*β* = −0.12, 95% CI = −0.27 to 0.03, *P* = 0.11). All coefficients highlighting the impact of the individual confounding variables are presented in Table S4. For both versions of the *N*-back task, larger effect estimates for the frequent and binge drinking group (compared to the low alcohol group) indicates stronger evidence of an association with deficits in WM performance, in comparison to effect sizes for the frequent drinking only group.

**Table 2.** Associations between alcohol use at age 15 years and *d′* at age 18 years for the 3-back task (*n* = 3319) in 50 multiply imputed datasets (standardized coefficients)

	*n* (%)	Model 1		Model 2		Model 3		Model 4	
		*β* (95% CI)	*P*	*β* (95% CI)	*P*	*β* (95% CI)	*P*	*β* (95% CI)	*P*
Low alcohol use		ref		ref		ref		ref	
Frequent drinking only	399 (11.8)	−0.02 (−0.16, 0.11)	0.74	−0.04 (−0.18, 0.08)	0.50	−0.04 (−0.17, 0.09)	0.57	0.02 (−0.11, 0.15)	0.75
Frequent and binge	354 (10.5)	−0.25 (−0.39, −0.11)	<0.001	−0.22 (−0.36, −0.08)	0.001	−0.23 (−0.37, −0.09)	0.001	−0.12 (−0.27, 0.03)	0.11

Model 1: unadjusted; Model 2: adjusted for sex, income, social economic position, maternal education, housing tenure, parity and maternal smoking in pregnancy; Model 3: further adjusted for working memory assessed at approximately age 11 years, and head injury/unconsciousness up to age 11 years; and Model 4: further adjusted for young person cigarette and cannabis use assessed at age 15 years.

Given the strength of the association between frequent and binge drinking and performance on the 3-back task, we examined whether frequent and binge drinking compared to consuming low amounts of alcohol was associated with the specific indices of WM functioning (Table 3). There was evidence to suggest that frequent and binge drinking was associated with the number of false alarms: (a) unadjusted model (*β* = −0.26, 95% CI = −0.44 to −0.07, *P* = 0.01); (b) model adjusted for sociodemographic confounders (*β* = −0.42, 95% CI = −0.42 to −0.05, *P* = 0.01) and (c) model further adjusted for WM at age 11 years and participants who had experienced a head injury/unconsciousness before age 11 years (*β* = −0.22, 95% CI = −0.41 to −0.22, *P* = 0.02). However, this association was attenuated when further adjusted for weekly cigarette smoking and cannabis use at age 15 years (*β* = −0.08, 95% CI = −0.26 to 0.11, *P* = 0.39). Models examining these associations for the 2-back are presented in Supplementary Material Table S5.

**Table 3.** Associations between frequent and binge drinking (compared to low alcohol users) at age 15 years and WM indices at age 18 years for the 3-back task (*n* = 3319) in 50 multiply imputed datasets

	Model 1		Model 2		Model 3		Model 4	
	*β* (95% CI)	*P*	*β* (95% CI)	*P*	*β* (95% CI)	*P*	*β* (95% CI)	*P*
Number of hits	−0.15 (−0.28, −0.01)	0.05	−0.13 (−0.27, 0.01)	0.06	−0.13 (−0.26, 0.00)	0.05	−0.10 (−0.25, 0.06)	0.22
Number of false alarms	−0.26 (−0.45, −0.05)	0.002	−0.23 (−0.41, −0.04)	0.005	−0.22 (−0.40, −0.04)	0.004	−0.09 (−0.28, 0.11)	0.30
Reaction time-hits	−0.03 (−0.17, 0.12)	0.73	0.00 (−0.14, 0.14)	0.99	−0.00 (−0.14, 0.14)	0.98	0.07 (−0.10, 0.23)	0.42
Reaction time-false alarms	0.01 (−0.14, 0.16)	0.91	0.04 (−0.11, 0.19)	0.64	0.03 (−0.12, 0.18)	0.68	0.11 (−0.06, 0.27)	0.23

Model 1: unadjusted; Model 2: adjusted for sex, income, social economic position, maternal education, housing tenure, parity and maternal smoking in pregnancy; Model 3: further adjusted for working memory assessed at approximately age 11 years, and head injury/unconsciousness up to age 11 years; and Model 4: further adjusted for young person cigarette and cannabis use assessed at age 15 years.

### Sensitivity analyses

Including the backward digit span at age 8 years (Supplementary Material Table S6a and b) produced almost identical results demonstrating evidence of an association for frequent and binge drinking and both the 2- and 3-back tasks (stronger associations for the 3-back task) compared to low alcohol users. Repeating the analyses using participants who had complete data on alcohol use, WM measures and all confounding variables produced weaker associations compared to the analyses using the fully imputed data (Supplementary Material Table S7a and b). Notably, the estimates for the fully adjusted models (Model 4) were similar.

## DISCUSSION

In this study, we found weak evidence of a prospective association between alcohol use at age 15 years and impaired WM performance 3 years later in a general population birth cohort. This association was evident in adolescents who were frequent and binge drinkers for the more demanding 3-back version of the task (assessed using the *d*′) after adjusting for a number of sociodemographic confounding variables, measure of WM at age 11 years and participants who had a head injury/unconsciousness before age 11 years. However, this association was attenuated when controlling for measures of cigarette smoking and cannabis use. When examining specific indices of WM, false alarms showed the strongest association, suggesting that performance on the task was affected by poor accuracy in rejecting non-targets rather than poor accuracy in detecting targets. There was insufficient evidence for an association between moderate drinking practices (i.e. frequent drinking only) and WM performance 3 years later for either the 2- or 3-back versions.

### Limitations

The present study should be considered in light of a number of limitations. First, the ALSPAC cohort suffers from attrition, which is higher among the socially disadvantaged (Wolke *et al.*, 2009). We attempted to minimize the impact of attrition using sensitivity analyses. Missingness was related to WM at age 11 years, alcohol use at age 15 years and sociodemographic variables. However, the results from the sensitivity analysis suggest that the pattern of missing data did not lead to biased effect estimates. Although we did observe differences in auxiliary measures depending on data availability, the direction and magnitude of the associations were consistent with the weighted models. Second, although alcohol use was self-reported, there is evidence to suggest that self-reported alcohol use is a reliable and valid method (Del Boca and Darkes, 2003). Third, as it cannot be ruled out that our findings could be over- or underestimating alcohol use, the inclusion of mutually exclusive alcohol measures ranging in order of severity helped to provide a more accurate account of adolescent drinking practices. Further, focusing on heavy drinking practices among adolescents, rather than more normative aspects of drinking such as frequency of drinking episodes, enables us to examine the hypothesized association in a more robust manner, as it has been suggested that focusing on frequency of drinking episodes may not be extreme enough to adversely impact WM (Khurana *et al.*, 2013). Fourth, an *N*-back measure assessed prior to alcohol initiation would have been optimal, however, the inclusion of the Counting Span task assessed at age 11 years demonstrate a robust pattern of results.

Fifth, although there is some debate in the literature surrounding the construct validity of performance on the *N*-back task as an indicator of WM ability, it has been argued that by using *N*-back performance indices from a signal-detection framework (i.e. d′) may reveal clearer insights about its validity as a measure of WM performance (Kane *et al.*, 2007; Meule, 2017; Haatveit *et al.*, 2010). Sixth, it is also possible that a number of higher order functions could influence this relationship since important maturational changes in brain organization and function continue well into late adolescence. For example, alcohol induced damage to the PFC and hippocampus could increase impulsive behaviour (Finn, 2002), lead to poor decision making (Crews and Boettiger, 2009) and motivation (Chambers *et al.*, 2003).

Finally, as we examined one potential causal pathway, it is possible that the direction of the association could work in both ways, that is impairments in WM may precede (and increase the risk of developing) alcohol problems (Peeters *et al.*, 2014). We were however able to include a number of measures to maximize the robustness of our findings: (a) ascertain the time order of exposure and outcome in our study, enabling the potential temporal associations between alcohol use and WM to be examined; (b) controlling for a measure of WM prior to the onset of alcohol use and participants who had a head injury helped to remove the possibility of deficits in WM influencing alcohol use and (c) although we cannot exclude the possibility of residual confounding, we have made adjustment for a number of confounding variables, including weekly smoking status, which was shown to have the strongest association with WM performance. Future work aims to follow up large prospective cohorts should take the possibly of reverse causality into account by including measures of alcohol use and WM at every assessment wave.

### Comparison with previous studies

To the best of our knowledge, this is the largest study to date to assess the prospective relationship between alcohol use and WM in adolescents. Our findings are consistent with the majority of research from neuropsychological and brain imagings that have demonstrated deficits in WM functioning in adolescents exhibiting problematic patterns of alcohol use (Squeglia *et al.*, 2009; Squeglia *et al.*, 2012; Peeters *et al.*, 2014). In terms of findings from community samples, a longitudinal cohort study of Dutch adolescents (aged 11–19 years) found no evidence of an association between heavy drinking in adolescence and maturation of executive functioning (Boelema *et al.*, 2015). The contrast in findings could be due to a number of possibilities. First, our study used the *N*-back task as opposed to the use of WM measured with the Amsterdam Neuropsychological Task (de Sonneville, 1999). Second, Boelema and colleagues examined change in WM performance across adolescence (examining maturation), while in our study WM performance was assessed at age 18 years (which is generally regarded as when WM matures). Finally, WM performance was measured in reaction times only, as opposed to the more comprehensive approach used in our study (e.g. identifying the correct number of hits, number of false alarms, discriminability index, and mean reaction times for hits and false alarms).

The inclusion of tobacco and cannabis use had a sizable impact on associations between alcohol use and WM. This is perhaps unsurprising as there is substantial evidence from animal studies linking cannabis use in adolescent with deficits in WM performance (Rubino *et al.*, 2009; Renard *et al.*, 2014; Verrico *et al.*, 2014). The association is further complicated as nicotine withdrawal has been shown to be associated with reductions in WM efficiency in animal studies (Levin *et al.*, 1990; Levin *et al.*, 2006). Evidence from human studies reveals a similar pattern of findings for adolescent tobacco and cannabis use on WM performance (Ilan *et al.*, 2004; Jacobsen *et al.*, 2005; Harvey *et al.*, 2007; Jacobsen *et al.*, 2007; Hanson *et al.*, 2010; Musso *et al.*, 2007).

Given that cannabis and tobacco use at the same time is popular among adolescents (Amos *et al.*, 2004), it is of interest to try to disentangle the independent and combined effects. A recent study examining the independent and combined impact of cannabis and nicotine on WM performance suggested that WM performance decreased with acute cannabis use and increased with tobacco use, while cannabis use was not associated with diminished WM when used with tobacco, suggesting that tobacco use may compensate for deficits in WM from cannabis (Schuster *et al.*, 2016).

## IMPLICATIONS AND CONCLUSIONS

Our findings contribute to the understanding of the relationship between alcohol use and WM in adolescents, and provide evidence that regular binge drinking in mid-adolescence is associated with impaired WM 3 years later, after adjusting for confounding variables. These findings have clinical and public health implications. For example, interventions aimed at preventing alcohol use in adolescents (Koning *et al.*, 2009) might be effective in reducing impairments in WM. In particular, a combined parent and student intervention was the most effective in reducing the onset of weekly alcohol use and frequency of drinking. One advantage is that interventions can yield beneficial effects on alcohol-related outcomes for adolescents even when delivered at young ages (Tanner-Smith and Lipsey, 2015). Although it is difficult to quantify the meaning of the deficit in WM in practical terms, deficits in WM have been shown to be related to academic achievement (Gathercole *et al.*, 2004), and impulsivity and risk-taking behaviours (Khurana *et al.*, 2013; Khurana *et al.*, 2015) in adolescents. Given the impact that cigarette and cannabis use had on the association between alcohol use and WM, it may be important to include these in future studies. Future research should explore possible mechanisms underlying this association and examine whether these associations persist into adulthood.

## Supplementary Material

Supplementary Data
