# Corrigendum: Alcohol Use in Adolescence and Later Working Memory: Findings From a Large Population-Based Birth Cohort

**DOI:** 10.1093/alcalc/agy005

**Published:** 2018-02-13

**Authors:** Liam Mahedy, Matt Field, Suzanne Gage, Gemma Hammerton, Jon Heron, Matt Hickman, Marcus R Munafò

*Alcohol and Alcoholism*, 2018: 1–8, doi: 10.1093/alcalc/agx113

The Funding Statement in the manuscript Alcohol Use in Adolescence and Later Working Memory: Findings From a Large Population-Based Birth Cohort (doi: 10.1093/alcalc/agx113) was incomplete on publication.

The Funding statement in its entirety should read:

The UK Medical Research Council and Wellcome Trust (Grant ref: 102215/2/13/2) and the University of Bristol provide core support for the Avon Longitudinal Study of Parents and Children (ALSPAC). This publication is the work of the authors and Jon Heron and Marcus R. Munafò will serve as guarantors for the contents of this paper. The work was undertaken with the support of the Medical Research Council and Alcohol Research UK (MR/L022206/1).

Furthermore, there was a missing decimal place in Table 2 of the same paper. The lower bound confidence interval for Frequent drinking only in Model 2 was given as −0.04 (−18, 0.08), while the correct format is −0.04 (−0.18, 0.08).

The author apologizes for these errors.

Here the online version of the paper has been corrected.

