# Supplementary material for: Alcohol Use in Adolescence and Later Working Memory: Findings From a Large Population-Based Birth Cohort
Source: Alcohol Alcohol. 2018 Jan 10;53(3):251–8. doi: 10.1093/alcalc/agx113 (PMC5913665; doi:10.1093/alcalc/agx113)
Supplement: Supplementary Data [file agx113supportingmaterial.docx]

13,954 live born offspring who survived to one year – 47.8% female

9,432 participants invited to clinic (mean age: 17y 10m) – 50.2% female

4,827 participants attended clinic – 56.1% female

3,721 participants had available data – 57.3% female

3,319 participants had available data for the 3-back task – 56.4% female (**sample used in analyses**)

3,351 participants had available data for the 2-back task – 56.9% female (**sample used in analyses**)

2,632 participants had available data for the 3-back task and alcohol measures – 56.6% female

2,677 participants had available data for the 2-back task and alcohol measures – 56.5% female

1,919 participants had complete data on outcome, exposures and confounders – 56.3% female

1,896 participants had complete data on outcome, exposures and confounders – 56.3% female

Figure S1. Sample attrition in ALSPAC

**Working memory – N-back task**

Participants continuously monitored a series of numbers presented on a computer screen, and pressed ‘1’ if the number was the same as the number presented N numbers ago, or ‘2’ if it was not. Stimuli were numbers 0–9, presented in black on white background with a random spatial jitter of 180 pixels in y-axis and 200 pixels in x-axis. Each target was presented for 500 ms, followed by a 3,000 ms response window. The practice block consisted of 12 trials containing two targets. Each experimental block was randomized and consisted of 48 trials, containing eight targets, with a single block for each of the 2- and 3-back conditions. In the 2-back condition, the target was the number that was identical to the one presented 2 trials back; while in the 3-back condition was identical to the one presented 3 trials back.

**Working memory – counting span task**

Children were presented with a number of red and blue dots on a white screen and asked to count the red dots out loud. The child was asked to recall the number of red dots seen on each screen with that set in the order they were presented. There were two practice sets and three sets with displays ranging from 2 to 5 screens. A total of 12 blocks were completed with three per load level.

**Inverse probability weighting**

Weights were derived from logistic regression models using variables associated with nonresponse, including maternal age, grandmother having a history of severe depression, maternal alcohol use in pregnancy, financial problems, maternal cannabis use and affordability problems. There was evidence of an association between all of the early-life exposures to adversity and loss to follow-up comparing complete cases and WM outcome data for all participants (*n*=9,432) invited to the clinic. We weighted the included respondents by the inverse of the probability of attending and used the Hosmer-Lemeshow test to assess model fit.

**Table S1. Selective attrition for working memory performance at age 18 years**

|  |  | 2-back |  |  | 3-back |  |
| --- | --- | --- | --- | --- | --- | --- |
|  | Available | Not available |  | Available | Not available |  |
|  | *n* (%) | *n* (%) | OR (95% CI) | *n* (%) | *n* (%) | OR (95% CI) |
| **Gender:** |  |  |  |  |  |  |
| Males | 1,408 (43.7) | 3,163 (54.4) | 0.65 (.60, .71) | 1,384 (43.7) | 3,187 (54.4) | 0.65 (.60, .71) |
| **Income:** |  |  |  |  |  |  |
| Low 20% | 351 (12.3) | 933 (21.8) | ref | 349 (12.4) | 935 (21.6) | ref |
| 40% | 512 (17.9) | 884 (20.6) | 0.65 (.55, .77) | 506 (17.9) | 890 (20.6) | 0.65 (.56, .77) |
| 60% | 559 (19.5) | 836 (19.5) | 0.56 (.48, .66) | 553 (19.6) | 842 (19.5) | 0.57 (.48, .67) |
| 80% | 679 (23.7) | 834 (19.5) | 0.46 (.39, .54) | 665 (23.5) | 848 (19.6) | 0.47 (.41, .56) |
| Highest % | 764 (26.7) | 798 (18.6) | 0.39 (.34, .46) | 752 (26.6) | 810 (18.7) | 0.40 (.34, .47) |
| **Maternal education:** |  |  |  |  |  |  |
| <O level | 1,567 (50.1) | 1,726 (33.2) | ref | 1,543 (50.1) | 1,750 (33.4) | ref |
| O level | 1,048 (33.5) | 1,826 (35.1) | 1.58 (1.42, 1.75) | 1,035 (33.6) | 1,839 (35.1) | 1.57 (1.41, 1.74) |
| >O level | 513 (16.4) | 1,646 (31.7) | 2.91 (2.58, 3.29) | 505 (16.4) | 1,654 (31.6) | 2.89 (2.56, 3.25) |
| **Social**: |  |  |  |  |  |  |
| iv-v | 102 (3.4) | 301 (6.3) | ref | 102 (3.4) | 301 (6.2) | ref |
| iii | 890 (29.5) | 1,935 (40.4) | 0.74 (.58, .93) | 871 (29.3) | 1,954 (40.4) | 0.76 (.60, .97) |
| ii | 1,420 (47.1) | 1,995 (41.6) | 0.48 (.38, .60) | 1,403 (47.2) | 2.012 (41.6) | 0.49 (.38, .61) |
| Professional | 601 (20.0) | 564 (11.8) | 0.31 (.25, .41) | 596 (20.1) | 569 (11.8) | 0.32 (.25, .42) |
| **Parity:** |  |  |  |  |  |  |
| First | 1,518 (48.5) | 2,339 (43.4) | ref | 1,496 (48.5) | 2,361 (43.4) | ref |
| Second | 1,113 (35.6) | 1,910 (35.4) | 1.11 (1.01, 1.22) | 1,099 (35.7) | 1,924 (35.4) | 1.11 (1.01, 1.22) |
| Third+ | 497 (15.9) | 1,147 (21.3) | 1.50 (1.32, 1.69) | 488 (15.8) | 1,156 (21.3) | 1.50 (1.33, 1.70) |
| **Tenure:** |  |  |  |  |  |  |
| Mortgaged | 2,719 (86.7) | 3,903 (72.0) | ref | 2,679 (86.8) | 3,943 (72.1) | ref |
| Private rent | 215 (6.9) | 589 (10.9) | 1.91 (1.62, 2.25) | 212 (6.9) | 592 (10.8) | 1.90 (1.61, 2.23) |
| Sub rent | 201 (6.4) | 928 (17.1) | 3.21 (2.74, 3.78) | 196 (6.4) | 933 (17.1) | 3.23 (2.75, 3.80) |
| **Maternal smoking:** |  |  |  |  |  |  |
| Yes | 341 (11.4) | 1,116 (22.0) | 2.19 (1.92, 2.49) | 336 (11.4) | 1,121 (21.9) | 2.17 (1.90, 2.48) |
| **WM at age 11:** |  |  |  |  |  |  |
| Linear term | 3.51 (0.83) | 3.36 (0.86) | 1.23 (1.16, 1.31) | 3.51 (0.84) | 3.35 (0.85) | 1.23 (1.17, 1.31) |
| **Head injury:** |  |  |  |  |  |  |
| Yes | 121 (3.4) | 217 (3.3) | 1.01 (.80, 1.27) | 118 (3.3) | 220 (3.4) | 0.99 (.79, 1.24) |
| **Cigarette smoking:** |  |  |  |  |  |  |
| Yes | 187 (6.9) | 277 (15.0) | 0.42 (.35, .51) | 181 (6.8) | 283 (14.9) | 0.42 (.34, .51) |
| **Cannabis use:** |  |  |  |  |  |  |
| Yes | 66 (2.5) | 97 (5.4) | 0.44 (.32, .61) | 63 (2.4) | 100 (5.4) | 0.43 (.31, .59) |
| **Adolescent alcohol use:** |  |  |  |  |  |  |
| Low alcohol use | 2,191 (81.9) | 1,334 (74.6) | ref | 2,160 (82.1) | 1,365 (74.4) | ref |
| Frequent drinking only | 283 (10.6) | 197 (11.0) | 0.88 (.72,1.06) | 280 (10.6) | 200 (10.9) | 0.88 (.73, 1.07) |
| Frequent and binge | 203 (7.6) | 258 (14.4) | 0.48 (.39, .58) | 192 (7.3) | 269 (14.7) | 0.45 (.37, .55) |

Note: Maternal education: <O level indicating no qualification; O level: indicating completion of school examinations at age 16; and >O level: indicating completion of college or university education at or after age 18; SEP grouped into 4 categories: iv-v: unskilled or semiskilled manual; iii: skilled manual or nonmanual; ii: managerial and technical; and i: professional

**Table S2. Selective attrition for alcohol use at age 15 years and WM at age 18 years**

|  | 2-back task | | | 3-back task | | |
| --- | --- | --- | --- | --- | --- | --- |
|  | Available | Not available | OR (95% CI) | Available | Not available | OR (95% CI) |
|  | *n* (%) | *n* (%) |  | *n* (%) | *n* (%) |  |
| *Low alcohol use* | 2,191 (81.9) | 556 (76.3) | ref | 2,160 (82.1) | 587 (75.6) | ref |
| *Frequent drinking only* | 283 (10.6) | 76 (10.4) | 1.06 (.80, 1.39) | 280 (10.6) | 79 (10.2) | 1.04 (.80, 1.35) |
| *Frequent and binge* | 203 (7.6) | 99 (13.4) | 1.92 (1.49, 2.49) | 192 (7.3) | 110 (14.1) | 2.11 (1.64, 2.71) |

**Working memory performance at age 18 years**

Overall, participants performed better on the 2-back task compared to the 3-back task using all four metrics: indicated by higher *d´* scores on the 2-back version (M=1.78, SD=1.25) compared to the 3-back version (M=1.16, SD=1.03); recording more correct hits: 2-back version (M=0.72, SD=0.21) compared to the 3-back version (M=0.27, SD=0.22); fewer false alarms: 2-back version (M=0.80, SD=0.22) compared to the 3-back version (M=0.79, SD=0.17), whilst also recording quicker reaction times for hits: 2-back version (M=706.2, SD=236.3) compared to the 3-back version (M=744.2, SD=303.2); and false alarms: 2-back version (M=677.1, SD=211.9) compared to the 3-back version (M=702.7, SD=264.3).

**Cognitive measure: digit span task**

To assess backwards digit span, the fieldworker gave a series of numbers out loud and asked the participant to recall them backwards with no time for pause. There were two practise items and seven test items (each item having two trials). Each item had one more number to recall than the preceding item (item 1 had two digits, item 7 had eight digits). The test was discontinued if a participant scored 0 on both trials of any item. For each item, the child gets 1 point for each number sequence correctly recalled resulting in a range from 0 to 7.

**Table S3.** **Associations between alcohol consumption at age 15 years and potential confounding variables**

**(multivariable analysis)**

|  | *Low alcohol use Frequent drinking only Frequent and binge drinking* | | | | | | | | |  |
| --- | --- | --- | --- | --- | --- | --- | --- | --- | --- | --- |
|  | *Reference group* | | *OR* | *95% CI* | *Wald/ p* | *OR* | | *95% CI* | *Wald/ p* |  |
| **Gender:** |  |  |  |  |  |  | |  |  |  |
| Males | -  -  -  -  - | | 1.32 | 0.99, 1.78 | .08 | 0.96 | | 0.64, 1.42 | .70 |  |
| **Income:** |  |  |  |  |  |  | |  |  |  |
| Low 20% |  |  | 0.88 | 0.47, 1.67 |  | 0.89 | | 0.43, 1.84 |  |  |
| 40% |  |  | 1.19 | 0.65, 2.19 |  | 0.82 | | 0.39, 1.72 |  |  |
| 60% |  |  | 1.22 | 0.67, 2.23 |  | 0.94 | | 0.45, 1.96 |  |  |
| 80% |  |  | 1.36 | 0.73, 2.53 | .19 | 1.37 | | 0.65, 2.90 | .31 |  |
| Highest % | - | | ref |  | | ref |  | | |  |
| **Maternal education:** |  |  |  |  |  |  | |  |  |  |
| <0 level | -  - | | 0.99 | 0.68, 1.44 |  | 1.10 | | 0.67, 1.78 |  |  |
| 0 level |  |  | 0.88 | 0.50, 1.55 | .57 | 0.82 | | 0.42, 1.67 | .55 |  |
| >0 level | - | | ref |  | | ref |  | | |  |
| **Social**: |  |  |  |  |  |  | |  |  |  |
| iv-v | -  -  - | | 2.51 | 0.58, 10.82 |  | 0.58 | | 0.19, 1.74 |  |  |
| iii |  |  | 2.42 | 0.55, 10.51 |  | 0.74 | | 0.24, 2.25 |  |  |
| ii |  |  | 2.29 | 0.51, 10.28 | .93 | 0.46 | | 0.13, 1.56 | .51 |  |
| Professional | - | | ref |  | | ref |  | | |  |
| **Parity:** |  |  |  |  |  |  | |  |  |  |
| First | - | | ref |  | | ref |  | | |  |
| Second | -  - | | 1.44 | 1.04, 1.99 |  | 1.62 | | 1.06, 2.47 |  |  |
| Third+ |  |  | 1.17 | 0.73, 1.85 | .31 | 1.07 | | 0.59, 1.99 | .39 |  |
| **Tenure:** |  |  |  |  |  |  | |  |  |  |
| Mortgaged | - | | ref |  | | ref |  | | |  |
| Private rent | -  - | | 1.85 | 1.03, 3.32 |  | 1.44 | | 0.66, 3.13 |  |  |
| Sub rent |  |  | 0.67 | 0.27, 1.71 | .94 | 0.94 | | 0.38, 2.30 | .73 |  |
|  |  |  |  |  |  |  | |  |  |  |
|  |  |  |  |  |  |  | |  |  |  |
| **Maternal smoking:** |  |  |  |  |  |  | |  |  |  |
| Yes | - | | 0.74 | 0.42, 1.32 | .26 | 1.69 | | 0.96, 3.01 | .11 |  |
| **WM at age 11:** |  |  |  |  |  |  | |  |  |  |
| Linear term | - | | 1.09 | 0.90, 1.33 | .32 | 1.24 | | 0.97, 1.61 | .08 |  |
| **Head injury:** |  |  |  |  |  |  | |  |  |  |
| Yes | - | | 1.15 | 0.64, 2.07 | .68 | 0.69 | | 0.25, 1.86 | .51 |  |
| **Cigarette smoking:** |  |  |  |  |  |  | |  |  |  |
| Yes | - | | 2.33 | 1.45, 3.74 | <.001 | 3.54 | | 2.17, 5.79 | <.001 |  |
| **Cannabis use:** |  |  |  |  |  |  | |  |  |  |
| Yes | - | | 3.06 | 2.15, 4.36 | <.001 | 7.09 | | 4.62, 10.86 | <.001 |  |

Note: Maternal education: <O level indicating no qualification; O level: indicating completion of school examinations at

age 16; and >O level: indicating completion of college or university education at or after age 18; SEP grouped into 4

categories: iv-v: unskilled or semiskilled manual; iii: skilled manual or nonmanual; ii: managerial and technical; and i:

professional

**Table S4. Associations between alcohol use at age 15 years and *d'* at age 18 years for the 3-back task (*n*=3,319) in 50 multiply imputed datasets including covariates**

|  | *Low alcohol use* | *Frequent drinking only* | | | *Frequent and binge drinking* | | |
| --- | --- | --- | --- | --- | --- | --- | --- |
|  |  | *3-back d’* | | | *3-back d’* | | |
|  | *Reference* | *β* | *95% CI* | *p* | *β* | *95% CI* | *p* |
| **Alcohol use:** |  | 0.02 | -.11, .15 | .75 | -0.12 | -.27, .03 | .11 |
| **Gender:** |  |  |  |  |  |  |  |
| Males | - | 0.46 | -.02, .11 | .16 | .05 | -.02, .11 | .15 |
| **Income:** |  |  |  |  |  |  |  |
| Low 20% | - | ref |  |  | ref |  |  |
| 40% | - | 0.07 | -.06, .19 | .27 | .07 | -.05, .19 | .26 |
| 60% | - | 0.11 | -.01, .23 | .07 | 0.11 | -.01, .23 | .07 |
| 80% | - | 0.12 | .00, .25 | .05 | 0.13 | .00, .25 | .05 |
| Highest % | - | 0.14 | .01, .27 | .03 | 0.14 | .02, .27 | .03 |
| **Maternal education:** |  |  |  |  |  |  |  |
| <0 level | - | ref |  |  | ref |  |  |
| 0 level | - | -0.18 | -.26, -.10 | <.001 | -0.18 | -.26, -.10 | <.001 |
| >0 level | - | -0.23 | -.34, -.13 | <.001 | -0.23 | -.34, -.14 | <.001 |
| **Social**: |  |  |  |  |  |  |  |
| iv-v | - | ref |  |  | ref |  |  |
| iii | - | -0.03 | -.21, .16 | .77 | -.03 | -.21, .16 | .76 |
| ii | - | -0.00 | -.19, .18 | .97 | -.01 | -.19, .18 | .95 |
| Professional | - | 0.11 | -.09, .31 | .30 | .10 | -.10, .31 | .31 |
| **Parity:** |  |  |  |  |  |  |  |
| First | - | ref |  |  | ref |  |  |
| Second | - | 0.07 | -.00, .14 | .07 | .07 | .00, .14 | .05 |
| Third+ | - | 0.07 | -.02, .16 | .15 | .07 | -.02, .17 | .13 |
| **Tenure:** |  |  |  |  |  |  |  |
| Mortgaged | - | ref |  |  | ref |  |  |
| Private rent | - | 0.07 | -.06, .20 | .31 | .07 | -.06, .20 | .28 |
| Sub rent | - | -0.14 | -.29, .00 | .05 | -.15 | -.30, -.01 | .04 |
|  |  |  |  |  |  |  |  |
|  |  |  |  |  |  |  |  |
| **Maternal smoking:** |  |  |  |  |  |  |  |
| Yes | - | -0.07 | -.17, .04 | .21 | -.06 | -.17, .04 | .24 |
| **WM at age 11:** |  |  |  |  |  |  |  |
| Linear term | - | 0.13 | ,10, .16 | <.001 | .13 | .10, .16 | <.001 |
| **Head injury:** |  |  |  |  |  |  |  |
| Yes | - | 0.08 | -.07, .22 | .31 | 0.07 | -.07, .22 | .32 |
| **Cigarette smoking:** |  |  |  |  |  |  |  |
| Yes | - | -0.37 | -.49, -.25 | <.001 | -0.34 | -.47, -.22 | <.001 |
| **Cannabis use:** |  |  |  |  |  |  |  |
| Yes | - | 0.07 | -.02, .16 | .12 | .10 | .01, .19 | .03 |

Note: Maternal education: <O level indicating no qualification; O level: indicating completion of school examinations at age 16; and >O level: indicating completion of college or university education at or after age 18; SEP grouped into 4 categories: iv-v: unskilled or semiskilled manual; iii: skilled manual or nonmanual; ii: managerial and technical; and i: professional

**Table S5. Associations between frequent and binge drinking (compared to low alcohol users) at age 15 years and WM indices at age 18 years for the 2-back task (*n*=3,319) in 50 multiply imputed datasets**

|  | Model 1 | | Model 2 |  | Model 3 | | Model 4 | |
| --- | --- | --- | --- | --- | --- | --- | --- | --- |
|  | *b* (*95% CI)* | *p* | *b* (*95% CI)* | *p* | *b* (*95% CI)* | *p* | *b* (*95% CI)* | *p* |
| *Number of hits* | -.15 (-.30, .01) | .07 | -.13 (-.28, .03) | .12 | -.13 (-.28, .03) | .10 | -.06 (-.23, .11) | .50 |
| *Number of false alarms* | -.13 (-.27, .02) | .10 | -.14 (-.29, .03) | .15 | -.12 (-.28, .03) | .12 | -.02 (-.18, .14) | .87 |
| *Reaction time-hits* | .05 (-.09, .20) | .48 | .06 (-.09, .21) | .42 | -.06 (-.09, .21) | .42 | .10 (-.07, .27) | .24 |
| *Reaction time-false alarms* | .01 (-.14, .15) | .92 | .02 (-.13, .17) | .79 | .02 (-.13, .17) | .81 | .08 (-.09, .25) | .34 |

Model 1: unadjusted; Model 2: adjusted for sex, income, social economic position, maternal education, housing tenure, parity, and maternal smoking in pregnancy; Model 3: further adjusted for working memory assessed at approximately age 11 years, and head injury/unconsciousness up to age 11 years; Model 4: further adjusted for young person cigarette and cannabis use assessed at age 15 years

**Table S6a.** **Associations between alcohol use at age 15 years and *d'* at age 18 years for the 2-back task (*n*=3,351) in 50 multiply imputed datasets**

|  |  | Model 1 | | Model 2 | Model 3 | | Model 4 | |
| --- | --- | --- | --- | --- | --- | --- | --- | --- |
|  | *n (%)* | *b* (*95% CI)* | *p* | *b* (*95% CI) p* | *b* (*95% CI)* | *p* | *b* (*95% CI)* | *p* |
| *Low alcohol use* |  | ref |  | ref | ref |  | ref |  |
| *Frequent drinking only* | 397 (11.7) | -.00 (-.15, .14) | .96 | -.03 (-.16, .10) .76 | -.04 (-.17, .10) | .58 | .02 (-.11, .16) | .79 |
| *Frequent and binge* | 368 (10.8) | -.17 (.32, -.03) | .02 | -.15 (-.30, .00) .05 | -.14 (-.29, .01) | .06 | -.04 (-.19, .12) | .66 |

Model 1: unadjusted; Model 2: adjusted for sex, income, social economic position, maternal education, housing tenure, parity, maternal smoking in pregnancy; Model 3: further adjusted for WM at age 8 years and head injury/unconsciousness up to age 11 years; Model 4: further adjusted for young person cigarette and cannabis use assessed at age 15 years

**Table S6b.** **Associations between alcohol use at age 15 years and *d'* at age 18 years for the 3-back task (*n*=3,319) in 50 multiply imputed datasets**

|  |  | Model 1 | | Model 2 | Model 3 | | Model 4 | |
| --- | --- | --- | --- | --- | --- | --- | --- | --- |
|  | *n (%)* | *b* (*95% CI)* | *p* | *b* (*95% CI) p* | *b* (*95% CI)* | *p* | *b* (*95% CI)* | *p* |
| *Low alcohol use* |  | ref |  | ref | ref |  | ref |  |
| *Frequent drinking only* | 399 (11.8) | -.02 (-.16, .11) | .74 | -.04 (-18, .08) .50 | -.05 (-.18, .08) | .44 | .01 (-.12, .14) | .88 |
| *Frequent and binge* | 354 (10.5) | -.25 (-.39, -.11) | <.001 | -.22 (-.36, -.08) .001 | -.22 (-.36, -.08) | .002 | -.11 (-.26, .04) | .16 |

Model 1: unadjusted; Model 2: adjusted for sex, income, social economic position, maternal education, housing tenure, parity, maternal smoking in pregnancy; Model 3: further adjusted for WM at age 8 years and head injury/unconsciousness up to age 11 years; Model 4: further adjusted for young person cigarette and cannabis use assessed at age 15 years

**Table S7a. Associations between alcohol use at age 15 years and WM at age 18 years (2-back task) – complete cases (*n*=1,919)**

| **2-Back *d'*** | Model 1 | | Model 2 | | Model 3 | | Model 4 | |
| --- | --- | --- | --- | --- | --- | --- | --- | --- |
|  | β (*95% CI)* | *p* | β (*95% CI)* | *p* | β (*95% CI)* | *p* | β (*95% CI)* | *p* |
| *Low alcohol use (82.5%)* | ref |  | ref |  | ref |  | ref |  |
| *Frequent drinking only (10.8%)* | .07 (-.05, .19) | .27 | .07 (-.05, .19) | .27 | .01 (-.12, .14) | .90 | .01 (-.13, .15) | .91 |
| *Frequent and binge (6.8%)* | -.11 (-.25, .03) | .13 | -.11 (-.25, .03) | .13 | -.14 (-.30, .02) | .08 | -.10 (-.27, .08) | .29 |

Model 1: unadjusted; Model 2: adjusted for sex, income, social economic position, maternal education, housing tenure, parity, maternal smoking in pregnancy; Model 3: further adjusted for WM at age 11 years, and head injury/unconsciousness up to age 11 years; Model 4: further adjusted for young person cigarette and cannabis use assessed at age 15 years

**Table S7b. Associations between alcohol use at age 15 years and WM at age 18 years (3-back task) – complete cases (*n*=1,896)**

| **3-back *d'*** | Model 1 | | Model 2 | | Model 3 | | Model 4 | |
| --- | --- | --- | --- | --- | --- | --- | --- | --- |
|  | β (*95% CI)* | *p* | β (*95% CI)* | *p* | β (*95% CI)* | *p* | β (*95% CI)* | *p* |
| *Low alcohol use (82.6%)* | ref |  | ref |  | ref |  | ref |  |
| *Frequent drinking only (10.8%)* | .08 (-.04, .20) | .20 | .03 (-.10, .17) | .62 | .02 (-.13, .16) | .79 | .05 (-.10, .19) | .54 |
| *Frequent and binge (6.7%)* | -.16 (-.31, -.02) | .03 | -.16 (-.33, .00) | .05 | -.20 (-.36 -.03) | .02 | -.16 (-.35, .02) | .08 |

Model 1: unadjusted; Model 2: adjusted for sex, income, social economic position, maternal education, housing tenure, parity, maternal smoking in pregnancy; Model 3: further adjusted for WM at age 11 years, and head injury/unconsciousness up to age 11 years; Model 4: further adjusted for young person cigarette and cannabis use assessed at age 15 years
